# Development of a Microbial-Based Integrated Pest Management Program for *Helicoverpa* spp. (Lepidoptera: Noctuidae) and Beneficial Insects on Conventional Cotton Crops in Australia

**DOI:** 10.3390/insects6020333

**Published:** 2015-04-09

**Authors:** Robert K. Mensah, Alison Young, Leah Rood-England

**Affiliations:** Australian Cotton Research Institute, NSW Department of Primary Industries, Locked Bag 1000, Narrabri, NSW 2390, Australia; E-Mails: alison.young@dpi.nsw.gov.au (A.Y.); leah.rood-england@dpi.nsw.gov.au (L.R.-E.)

**Keywords:** *Aspergillus* sp., *Helicoverpa* spp., entomopathogenic fungus, integrated pest management (IPM), transgenic cotton, *Bacillus thuringiensis*

## Abstract

Entomopathogenic fungi, when used as a microbial control agent against cotton pests, such as *Helicoverpa* spp., may have the potential to establish and spread in the environment and to have an impact on both pests and beneficial insects. Information on the effect of entomopathogenic fungi on pests and beneficial insects is crucial for a product to be registered as a biopesticide. The effect of the entomopathogenic fungus BC 639 (*Aspergillus* sp.) against *Helicoverpa* spp. and beneficial insects (mostly predatory insects) was studied in the laboratory and in cotton field trials. The results show that when *Helicoverpa* spp. second instar larvae were exposed to increasing concentrations (from 10^2^ to 10^9^) of the entomopathogenic fungus BC 639, the optimum dose required to kill over 50% of the insects was 1.0 × 10^7^ spores/mL. In the field trials, the number of *Helicoverpa* spp. per metre on plots treated with 1.0 or 0.50 L/ha of BC 639 was the same as on plots treated with the recommended rate of the commercial insecticide, Indoxacarb. However, when plots were treated with 0.25 L/ha of BC 639, this was not as effective at controlling *Helicoverpa* spp. as 1.0 or 0.5 L/ha BC 639 or Indoxacarb. BC 639 had less effect on predatory insects when applied at lower rates (0.50 and 0.25 L/ha) than at higher rates (1.0 L/ha). Thus, BC 639 was more selective against predators when applied at lower rates than at the higher rate, but was also more selective than Indoxacarb. Thus, the ability of BC 639 to control *Helicoverpa* spp. effectively with a minimal effect on predatory insects indicates its potential for enhancing integrated pest management programs and to sustain cotton production.

## 1. Introduction

Cotton crops in Australia are attacked by a wide range of pests, the major ones being the two lepidopteran pests, *Helicoverpa armigera* (Hübner) and *Helicoverpa punctigera* (Wallengren) [1]. These two pests are responsible for the use of synthetic insecticides in the Australian cotton industry from the 1960s until 1996–1997 [2]. In 1996, transgenic cotton containing genes from *Bacillus thuringiensis* (Bt) were introduced and then taken up by 90% of Australian cotton farmers. This cotton expresses the toxins Cry1Ac (Ingard^®^) (in transgenic cotton from 1996) and Cry 1Ac + Cry2Ab (Bollgard II^®^) (in transgenic cotton from 2004). The use of transgenic cotton has reduced the impact of the major pest (*Helicoverpa* spp.) on cotton crops in Australia [3,4,5,6]. These transgenic cotton crops are toxic to *Helicoverpa* spp. and other lepidopteran pests when ingested [7]. Thus, the introduction of transgenic cotton crops has decreased the use of synthetic insecticides against *Helicoverpa* spp. by 75%–80% [6]. In contrast, 10% of cotton farmers in Australia still grow non-transgenic cotton and rely exclusively on the use of synthetic insecticides to manage *Helicoverpa* spp. For these non-transgenic crops, there is a need to control *Helicoverpa* spp. with synthetic insecticides early in the season, which inadvertently suppresses populations of beneficial insects and potentially causes outbreaks of secondary pests, such as green mirids (*Creontiades dilutus*), cotton aphids (*Aphis gossypii*) and green vegetable bugs (*Nezara viridula*). The negative impact of this practice was of great concern to the cotton industry [6,7,8] until the adoption of transgenic (Bt) cotton crops.

Many beneficial insects, particularly predatory insects and specialist parasitoids, have been recorded in cotton crops worldwide. The role of beneficial insects in regulating pests in cotton agro-ecosystems has become increasingly important [9,10,11,12,13]. Although these beneficial insects do not show tightly coupled dynamics with their prey, because their population dynamics are not solely dependent on target pests, they often provide effective control, particularly in agro-ecosystems with non-chemical insecticide regimes [10,11,14,15,16].

In Australia, cotton crops are often grown in remote areas that have very sparse natural vegetation [11,14] and that lay fallow for most of the year. With no natural refuges and food sources for the adult natural enemies of pests, beneficial insect populations decline quickly, thereby limiting natural biological control [11,14,17]. Presently, cotton farmers in Australia are inadvertently discriminating against the natural enemies of *Helicoverpa* spp. and other pests by exclusively using synthetic insecticides on monocultured crops to control *Helicoverpa* spp. on conventional cotton and to control sucking pests on transgenic (Bt) cotton.

*Helicoverpa* spp. are difficult to manage on conventional cotton crops: first, because of the lack of diversity and stability in the cotton agro-ecosystem; second, because *Helicoverpa* spp. are highly mobile and highly migratory and can rapidly infest cotton crops; and third, because their eggs can escape natural enemy attack, as the natural enemies may not be present or established in sufficiently high numbers [2,11,14,18,19]. These factors have encouraged the use of synthetic broad-spectrum insecticides.

As a result, cotton farmers require new pest control methods to manage *Helicoverpa* spp. and to complement integrated pest management (IPM) on conventional cotton crops. Entomopathogenic fungi are known to be important natural enemies of many pests of agricultural crops [20,21]. Over 750 different species of fungi have been identified to date; these are cosmopolitan organisms that have been isolated from soils and have infected insects around the world [22,23,24]. All stages of insects, from the egg, larva or nymph to adult, can be killed by fungal pathogens. Fungi, unlike other pathogens, such as viruses and bacteria, do not need to be ingested to infect and kill the host. The advantage of using fungi for biological control is that the fungi are self-generating within or on the surface of the plant and can potentially provide ongoing protection as the plant grows, with little effect on non-herbivorous predators [5]. Several species of entomopathogenic fungi have been developed as alternatives to synthetic insecticides because of the low risk to humans, low risk to the environment and low pest resistance [22]. However, in Australia, only two isolates of *Metarhizium anisopliae* are commercially available, for the control of plague locusts and sugar cane grubs.

The present study evaluated the efficacy of a naturally occurring entomopathogenic fungus, BC 639 (*Aspergillus* sp.), against *Helicoverpa* spp. populations and on retaining populations of beneficial insects (e.g., predatory beetles, predatory bugs, predatory lacewings and spiders) on commercial conventional cotton crops. The study objectives were: (1) to assess the dose-response of the BC 639 fungus on *Helicoverpa* spp. larvae; (2) to assess the efficacy of different application rates of the BC 639 formulated product; (3) to identify the optimum rate of the BC 639 formulated product needed to control *Helicoverpa* spp. populations; and (4) to assess the impact of the BC 639 formulated product on the natural enemies of the target pests.

## 2. Materials and Methods

### 2.1. Isolation and Formulation of the Entomopathogenic Fungus BC 639

The BC 639 fungus was originally isolated from an infected insect in a commercial cotton farm [13]. The methods of Bahar *et al.* [25] and Mensah and Austin [13] were followed for isolating the spores for culturing and for preparing the formulation. Using a haemocytometer, the spore concentration was estimated to be 1.0 × 10^7^. The conidial germination was determined from 100 spore counts, and 100% germination was observed [5].

### 2.2. Experiment 1: Dose-Response of *Helicoverpa* spp. Second Instar Larvae in the Laboratory to BC 639

The experiment was conducted in the laboratory at the Australian Cotton Research Institute in Narrabri, NSW, Australia, from 10 to 28 February 2006. *Helicoverpa armigera* larvae were cultured in the laboratory and used in the study at the second instar stage.

BC 639 was tested at 10^1^, 10^2^, 10^3^, 10^4^, 10^5^, 10^6^, 10^8^ and 10^9^ spores/mL. For each treatment dose, 10 *H. armigera* second instar larvae were placed in a 90-mm Petri dish lined with filter paper and sprayed separately. The larvae from each treatment were then each transferred into separate 45-mm Petri dishes containing a fresh 19-mm conventional cotton leaf disc on filter paper. The Petri dishes were sealed and placed in an incubator set at 26–28 °C. Therefore, each treatment had 10 larvae placed individually in 10 Petri dishes.

The larvae were checked on 1, 2 and 3 days after treatment (DAT), and the number of dead and alive larvae were recorded. The mortality for each treatment was calculated, and a dose-response curve was developed for the concentration of BC 639 (log spores/mL) per mortality (%) of *Helicoverpa* spp. using GraphPad Prism 6 (GraphPad Software, Inc. San Diego, CA, USA). The number of spores/mL required to cause 50% mortality was extrapolated from the dose-response curve.

### 2.3. Experiment 2: Efficacy of BC 639 against *Helicoverpa* spp. First and Second Instar Larvae in the Laboratory

The experiment was conducted in the laboratory at the Australian Cotton Research Institute in Narrabri, NSW, Australia. *Helicoverpa armigera* first and second instar larvae were used for the experiment. The spore concentration used for this study was 1.0 × 10^7^. The conidial germination was 100%.

BC 639 was formulated into an oil-based product by Becker Underwood Pty. Ltd., Australia. The product was evaluated at 1% v/v (1.0 L/ha), and water was used as the control treatment. First instar larvae were used in the first experiment, and second instar larvae were used in the second experiment. For each experiment and each treatment, one larva per plant was placed on a cotton leaf and sprayed until run-off. Each treatment used 40 cotton plants infested with 40 larvae. After spraying, each larvae was transferred into a 35-mL clear plastic container (P10M; Solo, Urbana, IL, USA) containing a soybean-based artificial diet. The number of dead larvae were counted and recorded daily until all of the larvae had pupated. The mortality (%) was calculated for each treatment.

### 2.4. Experiment 3: Efficacy of Different Rates of BC 639 on the Survival of *Helicoverpa* spp. and Beneficial Insects on Commercial Cotton Crops

The trial was conducted on a dryland, commercial, conventional cotton farm at Getta Getta near Goondiwindi in the Macintyre Valley in Queensland in the 2006–2007 growing season.

The following treatments were evaluated for their effect on *Helicoverpa* spp., predatory beetles, predatory bugs, predatory lacewings and spiders: (1) 1.0 L/ha BC 639; (2) 0.50 L/ha BC 639; (3) 0.25 L/ha BC 639; (4) 0.85 L/ha Indoxacarb; and (5) unsprayed (untreated) control. The treatment plots were arranged in a randomized complete block design with six replicates per treatment. Each replicated plot measured 8-m wide and 220-m long.

The first foliar treatment was applied on 31 January 2007 and the second on 25 February 2007. Foliar treatments were applied using a ground rig sprayer fitted with flat fan nozzles (3 nozzles/row of cotton) to achieve a droplet size of 200 µm and an application volume of 100 L/ha. The treatment was applied in the morning when the temperature was between 20 °C and 28 °C. The decision for when to apply the treatment was based on the IPM Guidelines and the economic threshold of 2.0 larvae per metre as recommended by CottonLogic [1].

Visual counts of *Helicoverpa* spp. eggs and larvae, predatory beetles, bugs, lacewings and spiders on cotton plants for each treatment were made at approximately weekly intervals. For each of the six treatment replicates, a randomly selected 1-m length of planted row was examined. The number of *Helicoverpa* spp., predatory beetles, predatory bugs, predatory lacewings and spiders per metre for each treatment was determined.

### 2.5. Experiment 4: Efficacy of Different Rates of BC 639 against *Helicoverpa* spp. and Beneficial Insects on Conventional Cotton Crops at Norwood in 2007–2008

The trial was conducted on a commercial conventional cotton farm at Norwood, near Moree. The trial was conducted from 16 November 2007 to 11 March 2008.

The following treatments were evaluated for their effect on *Helicoverpa* spp. eggs, very small and small, medium and large larvae and beneficial insects: (1) 1.0 L/ha BC 639; (2) 0.50 L/ha BC 639; (3) 0.25 L/ha BC 639 (4) 0.85 L/ha Indoxacarb; and (5) unsprayed (untreated) control. The treatment plots were arranged in a randomized complete block design with four replicates per treatment. Each replicated plot measured 40-m wide (equivalent to the number of rows) and 90-m long. A 40-m wide buffer separated each of the Indoxacarb-treated plots, fungus plots and unsprayed plots.

The number of insects were counted 24 h before treatment and then 3, 7 and 14 DAT for each spray application. Foliar treatments were applied on 30 January 2008 (first spray) and 25 February 2008 (second spray) using a ground rig sprayer fitted with flat fan nozzles (3 nozzles/row of cotton plants) to achieve a droplet size of 200 µm and an application volume of 100 L/ha. The treatments were applied in the morning when the temperature was between 20 °C and 28 °C. On each occasion, the treatments were applied using 100 L of water per hectare. The control plot was unsprayed.

Visual counts of *Helicoverpa* spp. eggs and larvae, predatory beetles, bugs, lacewings and spiders for each treatment were made at approximately weekly intervals. For each of the four treatment replicates, a randomly selected 1-m length of planted row was examined. The number of *Helicoverpa* spp., predatory beetles, predatory bugs, predatory lacewings and spiders per metre for each treatment was determined.

### 2.6. Data Analysis

All experimental data were analysed using repeated measures analysis of variance (v. 2.03; Graphpad Instat and Prism Software Inc., San Diego, CA, USA). Treatments and sample dates were the independent variables. Tukey-Kramer multiple comparison tests were used to compare the treatment means.

## 3. Results

### 3.1. Experiment 1: Dose-Response of *Helicoverpa* spp. Second Instar Larvae to BC 639 in the Laboratory at the Australian Cotton Research Institute

**Figure 1:**
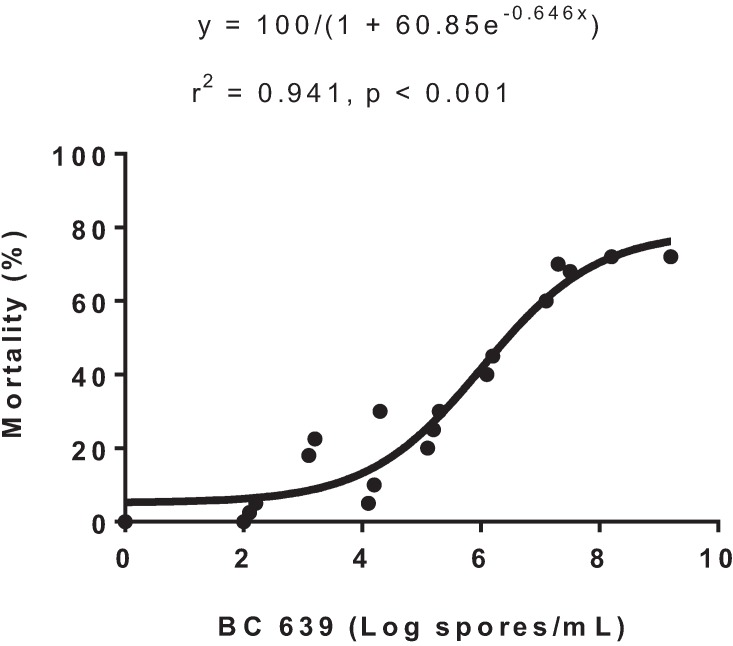
Dose-response curve for BC 639 and mortalities of *H. armigera* second instar larvae in the laboratory, 10–28 February 2006.

Exposure to progressively increasing concentrations of BC 639 from 10^2^ to 10^9^ spores/mL resulted in the deaths of *Helicoverpa* spp. second instar larvae (Figure 1). The optimum dose required to kill over 50% of the insects intercepts the curve at 10^7^ and was estimated to be 1.0 × 10^7^ spores/mL (Figure 1).

### 3.2. Experiment 2: Efficacy of BC 639 against *Helicoverpa* spp. First and Second Instar Larvae in the Laboratory

BC 639 caused 80% and 82% mortality of *H. armigera* first and second instar larvae, respectively, and was significantly different from the control (water)-treated larvae with 5% and 7.5% mortality, respectively (Table 1).

**Table 1:** Effect of BC 639 on *H. armigera* first and second instar larvae (*n* = 40 larvae/treatment) in the laboratory at the Australian Cotton Research Institute, Narrabri, Australia.

Treatments	% Mortality First Instar ± SE	% Mortality Of Second Instar ± SE
1% v/v BC 639 (*Aspergillus* sp.)	80.50 ± 5.62 ^a^	82.50 ± 5.47 ^a^
Water (control)	5.00 ± 3.44 ^b^	7.50 ± 4.10 ^b^

Means within columns followed by the same letters are not significantly different (*p* > 0.05; Tukey–Kramer multiple comparison test).

### 3.3. Experiment 3: Efficacy of Different Rates of BC 639 on the Survival of *Helicoverpa* spp. Eggs and Larvae on Conventional Cotton Crops at Getta Getta in 2006–2007

#### 3.3.1. *Helicoverpa* spp. Eggs

**Figure 2:**
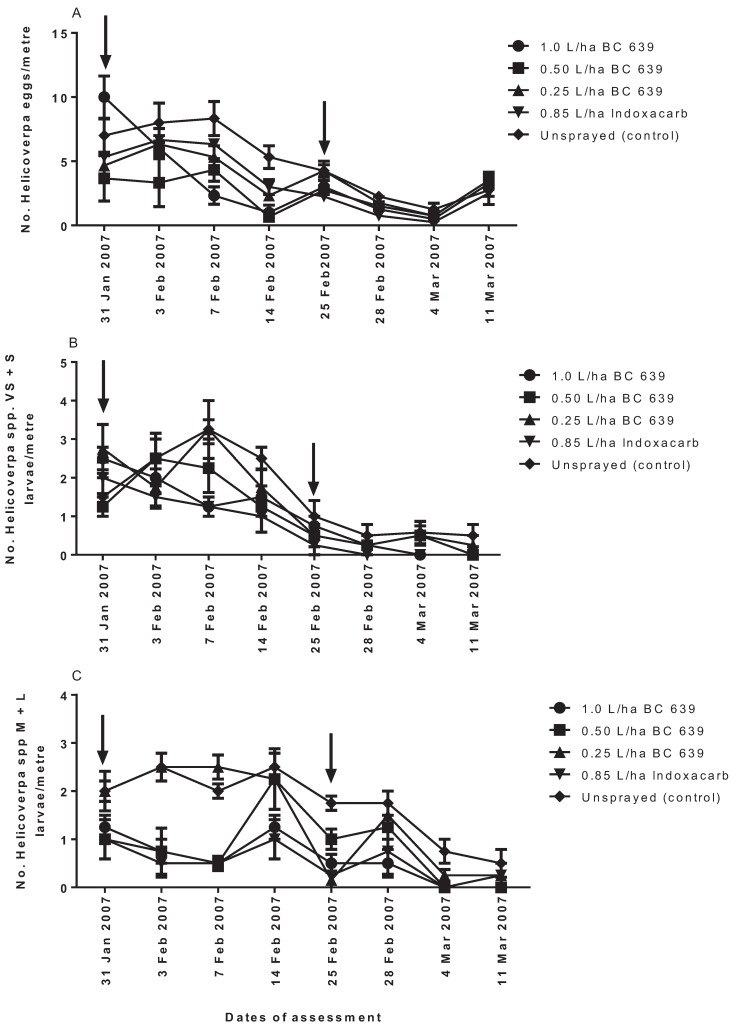
The efficacies of different rates of application of BC 639 and Indoxacarb in reducing numbers of *Helicoverpa* spp. (**A**) eggs, (**B**) very small + small (VS + S) larvae (*i.e*., instars 1–3) and (**C**) medium + large (M + L) larvae (*i.e.*, instars 4–6) per metre on commercial conventional cotton crops at Getta Getta near Goondiwindi, 2006–2007. The arrows indicate the date of treatment.

After the first spraying, oviposition by *Helicoverpa* spp. was the same among treated and control plots at 3 DAT, but was significantly lower (*p* < 0.003) on the BC 639- and Indoxacarb-treated plots than the unsprayed plots at seven and 14 DAT (Figure 2A). No significant difference (*p* > 0.05) was detected after the first spraying among cotton plants treated with different rates of BC 639 and Indoxacarb (Figure 2A).

After the second spraying, the number of *Helicoverpa* spp. eggs per metre on plots treated with BC 639 and 0.85 L/ha Indoxacarb was the same at 3, 7 and 14 DAT, but they were significantly lower (*p* < 0.02) than the unsprayed plots at three DAT (Figure 2A). Overall, there was no strong evidence that application of the fungus deterred egg laying by *Helicoverpa* spp. on treated cotton plants (Figure 2A).

#### 3.3.2. *Helicoverpa* spp. Very Small + Small (First to Third) Instar Larvae

Significant differences (*p* < 0.05) in the number of first and second instar larvae per metre were found among treatments at seven DAT (Figure 2B). Plots treated with 1.0 L/ha, 0.50 L/ha and 0.85 L/ha Indoxacarb recorded the lowest number of very small and small (VS + S) (first to third) instar larvae per metre after the first spraying (Figure 2B). No significant differences were detected between plots treated with 0.25 L/ha BC 639 and the control (unsprayed) plots after the first spraying (Figure 2B). The numbers of VS + S instar larvae per metre recorded on plots treated with 1.0 L/ha and 0.50 L/ha BC 639 was the same as plots treated with Indoxacarb after the first spraying. At 14 days after the first spraying, the number of VS + S larvae per metre was the same among treated and control plots (Figure 2B). In contrast, no significant differences were detected between the BC 639-treated plots and the control (unsprayed) plots (Figure 2B).

#### 3.3.3. *Helicoverpa* spp. Medium + Large (Fourth to Sixth) Instar Larvae

Following the first spraying, the number of medium + large (M + L) larvae was significantly lower (*p* < 0.01) on 1.0 L/ha and 0.50 L/ha BC 639- and 0.85 L/ha Indoxacarb-treated plots than plots treated with 0.25 L/ha BC 639 or the control (unsprayed) plots (Figure 2C). The number of M + L larvae per metre recorded on plots treated with 0.25 L/ha BC 639 was the same (*p* > 0.05) as the control (unsprayed) plots (Figure 2C), indicating that the 0.25 L/ha rate of BC 639 was not effective against *Helicoverpa* spp.

Following the second spraying, the same number of M + L larvae per metre (*p* > 0.05) was found among treatments at three and 14 DAT (Figure 2C). At seven DAT, however, the number of M + L recorded on the unsprayed plots was significantly higher (*p* < 0.03) than the BC 639- and Indoxacarb-treated plots. In contrast, no significant difference (*p* > 0.05) in the number of M + L larvae was found on 1.0 L/ha 0.50 and 0.25 L/ha BC 639-treated plots, Indoxacarb-treated plots and the unsprayed plots at 14 DAT (Figure 2C).

#### 3.3.4. Beneficial Insects

Beneficial insects found at the study site were mainly predatory insects (Table 2). Predators of *Helicoverpa* spp. identified from the treated plots were predatory beetles (only three species were predominant in the study site), bugs, lacewings and spiders (Table 2).

**Table 2:** Predators of cotton pests sampled and identified from the study sites in 2006–2008.

Order	Family	Species	Group
Coleoptera	Coccinellidae	*Coccinella transversalis* (Fabricius) *Diomus notescens* (Blackburn)	Predatory beetles
	Melyridae	*Dicranolaius bellulus* (Guerin-Meneville)	
Hemiptera	Nabidae	*Nabis capsiformis* (Germar)	Predatory bugs
	Lygaeidae	*Geocoris lubra* (Kirkaldy)	
	Pentatomidae	*Cermatulus nasalis* (Westwood) *Ochelia schellenbergii* (Guerin-Meneville) *Coranus triabeatus* (Horvath)	
Neuroptera	Chrysopidae	*Chrysopa* spp.	Predatory lacewings
	Hemerobiidae	*Micromus tasmaniae* (Walker)	
Araneidae	Lycosidae	*Lycosa* spp.	Spiders
	Oxyopidae	*Oxyopes* spp.	
	Salticidae	*Salticidae* spp.	
	Araneidae	*Araneus* spp.	

##### 3.3.4.1. Predatory Beetles

**Figure 3:**
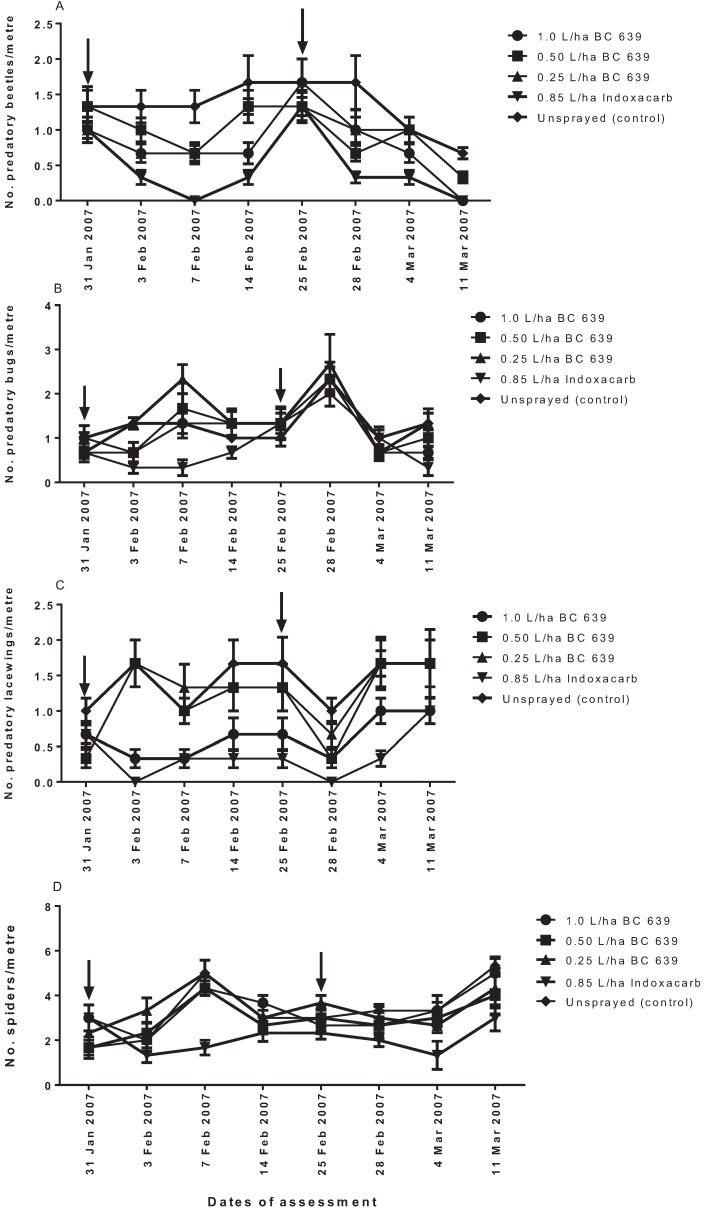
Efficacy of different rates of application of BC 639 and synthetic insecticide (Indoxacarb) on the number of (**A**) predatory beetles, (**B**) predatory bugs, (**C**) predatory lacewings and (**D**) spiders on commercial conventional cotton crops at Getta Getta near Goondiwindi, 2006–2007. The arrows indicate the date of treatment.

Following the first spraying on 31 January 2007, the number of predatory beetles per metre on the plots treated with 0.5 and 0.25 L/ha BC 639 (lower rates) was not significantly different (*p* > 0.05) from the unsprayed (control) plots, although plots treated with 1.0 L/ha BC 639 (higher rate) and Indoxacarb were significantly lower (*p* < 0.01) than the unsprayed plots at 3, 7 and 14 DAT (Figure 3A). The number of predatory beetles per metre on plots treated with 1.0 L/ha BC 639 was less than that on unsprayed plots (control), there were significantly more (*p* < 0.01) than on the Indoxacarb-treated plots (Figure 3A).

After the second spray, the number of predatory beetles per metre on the BC 639-treated plots was identical (*p* > 0.05) to the unsprayed plots at three and seven DAT, but was significantly lower (*p* < 0.01) than the unsprayed plots at 14 DAT (Figure 3A). In contrast, the number of predatory beetles per metre on the Indoxacarb-treated plots was significantly lower (*p* < 0.001) than the unsprayed plots at 3, 7 and 14 DAT (Figure 3A).

##### 3.3.4.2. Predatory Bugs 

Following the first spraying, the number of predatory bugs per metre on 1.0 and 0.5 L/ha BC 639-treated plots and Indoxacarb-treated plots was the same, but it was significantly lower (*p* < 0.01) than the 0.25 L/ha BC 639 and unsprayed (control) plots at three DAT (Figure 3B). However, at seven and 14 DAT, the number of predatory bugs per metre on the BC 639-treated plots (all rates) was the same as the unsprayed plots, but significantly higher (*p* < 0.001) than the Indoxacarb-treated plots (Figure 3B). The Indoxacarb-treated plots consistently showed fewer predatory bugs per metre after the first spraying (Figure 3B).

Following the second spraying, the number of predatory bugs per metre was not significantly different (*p* > 0.05) between the BC 639-treated plots, Indoxacarb-treated plots and the unsprayed plots (Figure 3B). There could have been an influx of bugs from the unsprayed and BC 639-treated plots into the Indoxacarb-treated plots or the effect could have been due to spray drift.

##### 3.3.4.3. Predatory Lacewings

The predominant predatory lacewings identified from the study plots were *Chrysopa* spp. and *Micromus tasmaniae* (Table 2). Following the first spraying, the number of predatory lacewings per metre on plots treated with 1.0 L/ha BC 639 and Indoxacarb was the same, but it was lower than 0.50 and 0.25 L/ha BC 639 and the unsprayed plots at 3 DAT (*p* < 0.0001), 7 DAT (*p* < 0.001), 14 DAT (*p* < 0.01) and 25 DAT (*p* < 0.001 (Figure 3C)). In contrast, plots treated with 0.50 and 0.25 L/ha BC 639 had the same number of predatory lacewings as the unsprayed plots (Figure 3C).

Following the second spraying, the number of lacewings per metre in plots treated with all rates of BC 639 and the unsprayed plots was the same at three DAT, but was significantly different (*p* < 0.01) from the Indoxacarb-treated plots (Figure 3C). At seven DAT, the number of predatory lacewings found on 0.5 and 0.25 L/ha and the unsprayed plots was the same (*p* > 0.05), and this was significantly different from the 1.0 L/ha (*p* < 0.01) and Indoxacarb-treated plots (*p* < 0.001) (Figure 3C). No significant difference (*p* > 0.05) was recorded among the BC 639 rates, Indoxacarb and the unsprayed plots at 14 DAT (Figure 3C).

##### 3.3.4.4. Spiders 

The spiders identified from the study plots were *Lycosa* spp., *Oxyopes* spp., *Salticidae* spp. and *Araneus* spp. (Table 2). The number of spiders per metre on plots treated with the BC 639 rates and the unsprayed plots was the same following the first (31 January 2007) and second (25 February 2007) treatments (Figure 3D). However, the number of spiders per metre on the Indoxacarb-treated plots following the first and second treatments was significantly lower (p < 0.01) than the BC 639 and unsprayed plots at seven DAT after the first and second spraying (Figure 3D).

### 3.4. Experiment 4: Efficacy of Different Rates of BC 639 against Helicoverpa spp. and Beneficial Insects on Conventional Cotton Crops at Norwood in 2007–2008

#### 3.4.1. *Helicoverpa* spp. Eggs

**Figure 4:**
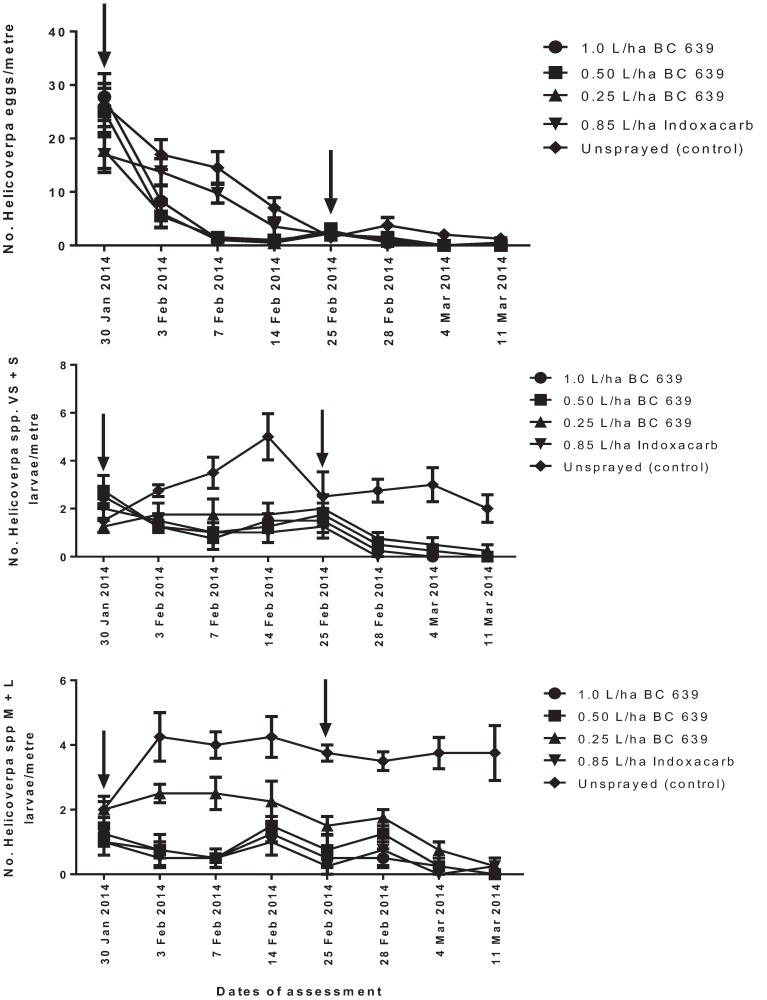
The efficacies of different rates of application of BC 639 and synthetic insecticide (Indoxacarb) on reducing the numbers of *Helicoverpa* spp. (**A**) Eggs, (**B**) very small + small (VS + S) larvae (*i.e*., Instars 1–3) and (**C**) medium + large (M + L) larvae (*i.e*., Instars 4–6) per metre on commercial conventional cotton crops at Norwood near Moree, 2007–2008. The arrows indicate the date of treatment.

The first spraying was applied when the density of *Helicoverpa* spp. eggs ranged from 18 to 28 eggs per metre (Figure 4A). After the first spraying, the number of eggs recorded on plots treated with BC 639 rates, Indoxacarb and the unsprayed plots was the same at 3, 14 and 25 DAT (*p* > 0.05) (Figure 4A). However, the number of *Helicoverpa* spp. eggs per metre recorded on plots treated with 1.0 and 0.5 L/ha BC 639 and Indoxacarb was significantly lower (*p* < 0.01) at seven DAT than plots treated with 0.25 L/ha and unsprayed plots (Figure 4A). However, after the second spraying, the number of eggs per metre was the same among treated and unsprayed plots (Figure 4A).

#### 3.4.2. *Helicoverpa* Very Small and Small Larvae

After the first spraying, the plots treated with BC 639 rates and Indoxacarb had a significantly lower (*p* < 0.01) number of VS + S larvae at 3 (*p* < 0.0001), 7 (*p* < 0.003) and 14 DAT than the unsprayed plots (Figure 4B). Similar results were found for plots treated with BC 639 rates, Indoxacarb and the unsprayed plots at 3, 7 and 14 DAT (Figure 4B). After the second spray, the number of *Helicoverpa* VS + S larvae recorded on plots treated with BC 639 rates and Indoxacarb was the same and below the recommended threshold (two larvae per metre) used to manage these pests on conventional cotton crops [1].

#### 3.4.3. *Helicoverpa* Medium and Large Larvae

The number of *Helicoverpa* spp. M + L larvae recorded on plots treated with BC 639 rates and Indoxacarb after the first treatment was similar and significantly lower at 3 (*p* < 0.0005), 7 (*p* < 0.0001) and 14 DAT (*p* < 0.003) than the unsprayed plots (Figure 4C). Similarly, the number of M + L larvae per metre on plots treated with BC 639 and Indoxacarb was the same after the second spraying (Figure 4C). The unsprayed plots had the highest number of M + L larvae per metre at 3–14 DAT (3.50–3.75 per metre) (Figure 4C).

#### 3.4.4. Predatory Beetles

**Figure 5:**
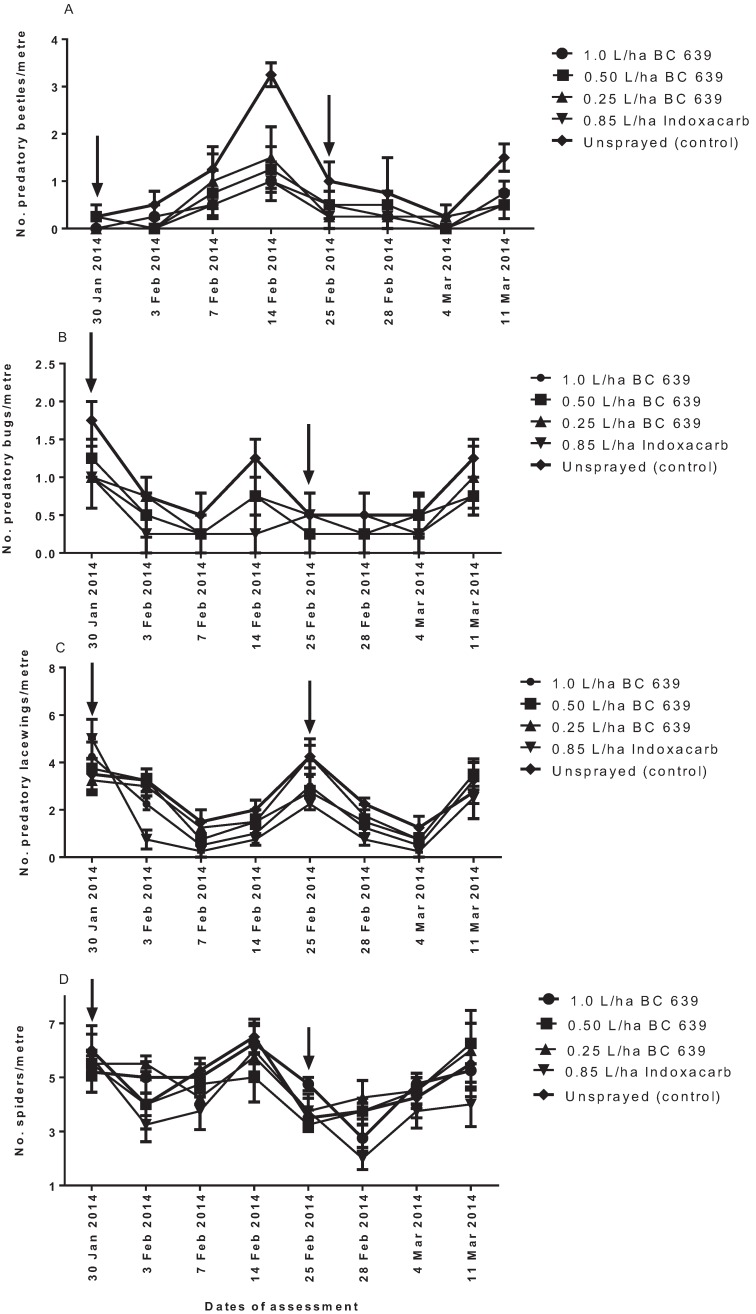
Numbers of (**A**) predatory beetles, (**B**) predatory bugs, (**C**) predatory lacewings and (**D**) spiders per metre on conventional cotton crops at Norwood near Moree in 2007–2008. Arrows indicate the dates of treatment application with BC 639 and Indoxacarb. Error bars represent the standard error of the mean.

Predatory insects identified from the study plots are listed in Table 2. The predominant predatory beetles identified from the study plots were *C. transversalis*, *D. notescens* and *D. bellulus* (Table 2), and the number of the predatory beetles per metre is given in Figure 5A. No significant differences (*p* > 0.05) were found among BC 639 rates, Indoxacarb and the unsprayed plots at three and seven DAT after the first spraying (Figure 5A). However, the number of predatory beetles per metre on the unsprayed plot (3.25 per metre) was significantly higher (*p* < 0.001) than that on the plots treated with BC 639 rates and Indoxacarb at 14 DAT (Figure 5A). Similarly, no significant differences were recorded in the number of predatory beetles per metre among treatments at three and seven DAT, but there were significantly different (*p* < 0.01) numbers between the unsprayed plots, the plots treated with BC 639 and Indoxacarb at 14 DAT after the second spraying (Figure 5A). The effect could have been a result of spray drift. The number of predatory beetles per metre on plots treated with Indoxacarb and BC 639 rates at 14 DAT was not significantly different (*p* > 0.05) (Figure 5A).

#### 3.4.5. Predatory Bugs

The predominant predatory bugs identified from the study plots are given (Table 2). No significant differences (*p* > 0.05) were found among the unsprayed plots, BC 639- and Indoxacarb-treated plots after the treatments (Figure 5B).

#### 3.4.6. Predatory Lacewings

The predominant predatory lacewings identified from the study plots were *Chrysopa* spp. and *Micromus tasmaniae* (Table 2). No significant differences were found among BC 639-treated plots and the unsprayed plots after the first and second treatments (Figure 5C). The Indoxacarb-treated plots consistently showed the lowest number of predatory lacewings per metre (about 50% lower than the BC 639-treated plots) throughout the study. However, it was only at three DAT since the first spray that the difference between the Indoxacarb-treated plots was significantly lower (*p* < 0.001) than the BC 639-treated plots and the unsprayed plots (Figure 5C). This indicates that BC 639 had minimal impact on predatory lacewings compared with Indoxacarb (Figure 5C).

#### 3.4.7. Spiders

The predominant spiders identified from the study plots are given (Figure 5D). No significant differences were found among the unsprayed plots, BC 639- and Indoxacarb-treated plots after the first and second treatments (Figure 5D).

## 4. Discussion

The study has shown that the application of a formulated product of BC 639 entomopathogenic fungus effectively controlled populations of *Helicoverpa* spp. on commercial cotton crops with minimal or no effect against predators of *Helicoverpa* spp. One exception occurred when the product was applied at 1.0 L/ha (the highest rate in this study), and some negative effect was evident on predatory insects at the Getta Getta study site. In our study, the fungus killed *Helicoverpa* spp. larvae within 3–7 DAT. Application of BC 639 at 1.0 and 0.50 L/ha reduced the number of *Helicoverpa* spp. per metre, and these rates were consistently more effective than the lower 0.25 L/ha rate. Thus, the optimum effective rate of application of BC 639 against *Helicoverpa* spp. on cotton crops to reduce pest damage was 0.50 L/ha. Similar results were obtained when BC 639 was applied to *Creontiades dilutus* on commercial conventional cotton crops [15].

According to [5,25,26], entomopathogenic fungi, such as BC 639, can negatively affect the survival of soft-bodied beneficial insects. In the present study, application of BC 639 at 0.5 and 0.25 L/ha did not significantly affect predatory beetle population, but when BC 639 was applied at 1.0 L/ha, it affected the number of predatory beetles per metre, with a similar effect to that of Indoxacarb (see Figure 3A). Although the application of 1.0 L/ha BC 639 reduced the number of predatory beetles per metre, the negative effect of the 1.0 L/ha was significantly less than the negative effect of Indoxacarb. The present results also showed that following the first spraying, 1.0 and 0.50 L/ha BC 639 had a negative effect on the predatory bugs at three DAT, but the population recovered at seven and 14 DAT. In contrast, BC 639 applied at 0.25 L/ha had no effect on the predatory bugs. In the case of predatory lacewings, the 1.0 L/ha BC 639 had a similar negative effect on predatory lacewings as the Indoxacarb treatment. Furthermore, Indoxacarb was consistently disruptive to predatory bugs and spiders, whereas BC 639 had no effect on the spiders. These results suggest that the application of lower rates of BC 639 had a more selective effect on predatory beetles, bugs, lacewings and spiders than the Indoxacarb. Thus, the number of predatory insects recorded for the lower rates of BC 639-treated plots and the unsprayed plots was the same and significantly higher than the 1.0 L/ha BC 639 rate, which prevents us from speculating that BC 639 is essentially benign at all rates on soft-bodied insects. In the case of Indoxacarb, our study found that the selectivity of Indoxacarb only occurred in the treated plots that were adjacent to the unsprayed plots, indicating a possible influx of predators from the unsprayed plots to the Indoxacarb-treated plots.

Entomopathogenic fungi in general are known to be important natural enemies of many pests of agricultural crops [20] and have an advantage over synthetic insecticides in the conservation of beneficial insects [20]. The BC 639 fungus used in this study is self-generating on the surface of the plant and can potentially provide ongoing protection as the plant grows, with little effect on non-herbivorous predators [15,5]. Cotton ecosystems contain many strains of fungi, such as in the soil and on plant debris, but long-term research is required to identify potential fungi and to increase its pathogenicity and adaptability to a particular environment, so as to enhance its ability to infect and control insects on agricultural crops.

The selectivity of BC 639 against predatory insects indicates that this fungus has the potential to enhance conservational biological control, to support IPM and to reduce synthetic insecticide use on conventional cotton crops. IPM involves using all means of managing pest populations with the aim of reducing insecticide use while maintaining profitability, yield and fibre quality [15].

Similar to many other cotton production systems worldwide, the Australian cotton cropping system is strictly monoculture. This practice and the use of synthetic insecticides inadvertently discriminates against beneficial arthropods. Therefore, there is a need to use biological pesticides, such as lower rates of BC 639, to conserve beneficial insects and support IPM in cotton agro-ecosystems. By integrating BC 639 fungal biopesticide with beneficial insects, rather than relying on beneficial insects alone, we can effectively enhance the control of *Helicoverpa* spp. in monoculture cropping systems, such as cotton. This is because *Helicoverpa* spp. are highly migratory and can quickly infest cotton crops and lay their eggs. Such behaviour by *Helicoverpa* spp. means that by the time beneficial insects arrive and establish in the cotton crops, the pest might have developed into medium and large larvae (fourth to sixth instar), which are too large for the beneficial insects (mostly predators) to feed on and, thus, to control. Hence, beneficial insects (particularly predatory insects) need to be well established in the cotton crops prior to the arrival of the pest (*Helicoverpa* spp.) to enable a sufficiently rapid response for population control. Thus, an IPM strategy developed against *Helicoverpa* spp. should involve tools and strategies that enhance the control of these pests; these could include fungal biopesticides, such as BC 639, which is selective against beneficial insects.

An IPM program for large-, medium- and small-scale monoculture crops, such as cotton, should develop in a stepwise progression that initially involves strategies to establish beneficial insects in the cotton field [13], followed by integration with IPM-compatible tools, such as the BC 639 entomopathogenic fungus that controls the target pest, but is selective for beneficial insects. Intervention with broad-spectrum synthetic pesticides should be used as a last resort when pests exceed thresholds and when no other effective selective management options are available.

The success of IPM programs in many cropping systems, especially conventional cotton systems, will depend on the availability of IPM-compatible tools and strategies to farmers. To adopt a true IPM program, farmers will need alternative pest control tools that have a minimal impact on the beneficial insects, whatever crop is grown. For cotton farmers in Australia and other parts of the world, the increase in *Helicoverpa* spp. populations on conventional cotton crops, yield loss, high cost of cotton production, insecticide resistance, disruption of beneficial species and environmental impacts are considered to be the major factors influencing the implementation of a sustainable IPM program for cotton crops. Thus, it is crucial that research continues to identify and develop new IPM-compatible tools and alternative strategies for managing and controlling *Helicoverpa* spp. and other pests [6,12,13]. Hence, the use of biological pesticides, including lower rates of BC 639 entomopathogenic fungus, is very important to support IPM in cotton agro-ecosystems.

The microbial biopesticide BC 639 is selective at lower application rates of 0.5 and 0.25 L/ha against predatory insects and has been used to effectively control *Helicoverpa* spp. on commercial cotton crops.

## 5. Conclusions

Application of a formulated entomopathogenic fungus (*Aspergillus* sp. BC 639) effectively controlled populations of *Helicoverpa* spp. on commercial cotton crops with minimal or no effect against predators of *Helicoverpa* spp. when the product was applied at 0.5 and 0.25 L/ha (the lower rates in this study). The fungus killed *Helicoverpa* spp. larvae within 3–7 DAT. Application of BC 639 at 1.0 and 0.50 L/ha was more effective than applications at the lower rate of 0.25 L/ha. The 1.0 and 0.50 L/ha rates of BC 639 had a similar efficacy against *Helicoverpa* spp. larvae as the 0.85 L/ha treatment with the commercial conventional synthetic insecticide Indoxacarb. This result suggests that the optimum effective rate for the application of BC 639 to control *Helicoverpa* spp. infestations on cotton crops was 0.50 L/ha. The microbial biopesticide BC 639 was selective against predatory insects at lower rates, and it can effectively control *Helicoverpa* spp. on commercial cotton crops and, thus, has a great potential to complement IPM programs in cotton crops.
